# Late In-Stent Thrombosis After Carotid Artery Stenting for Symptomatic Internal Carotid Artery Stenosis in a Patient With JAK2 V617F-Positive Essential Thrombocythemia: An Illustrative Case Report

**DOI:** 10.7759/cureus.47688

**Published:** 2023-10-25

**Authors:** Mika Arai, Kenta Nakase, Fumihiko Nishimura, Ichiro Nakagawa, Hiroyuki Nakase

**Affiliations:** 1 Neurosurgery, Nara Medical University, Kashihara, JPN; 2 Neurosurgery, Nara Medical Unversity, Kashihara, JPN; 3 Neurosurgery, Heisei Memorial Hospital, Kashihara, JPN

**Keywords:** in-stent thrombosis, carotid artery stenting, jak2 v617f, essential thrombocythemia, internal carotid artery stenosis

## Abstract

Essential thrombocythemia (ET) is a myeloproliferative disorder complicated by thrombosis in 13% of cases. The Janus kinase *2 (JAK2)* V617Fmutation is present in 60% of ET cases, and it has recently been reported that the mutation itself is a significant contributor to ischemic stroke. Here, we present an illustrative case of late in-stent thrombosis following carotid artery stenting (CAS) in a patient with ET and* the JAK2* V617F mutation presenting with symptomatic internal carotid artery (ICA) stenosis. An 80-year-old man with a history of *JAK2 *V617F-positive ET suffered from left upper motor weakness and numbness. Magnetic resonance imaging/magnetic resonance angiography revealed multiple acute cerebral infarctions scattered in the right frontal and parietal lobes and right ICA stenosis. Despite continued antiplatelet therapy, plaque size did not decrease. CAS was performed one month later; however, five months after the procedure, in-stent thrombus growth was observed, leading to severe stenosis despite administering antiplatelet or anticoagulant drugs. The thrombus was eventually resolved with increased doses of hydroxyurea and aspirin administration. In conclusion, controlling platelets and inflammation with hydroxyurea and aspirin may help improve the condition in case of rapid thrombosis due to the *JAK2* V617F mutation, unlike other thromboses. This case highlights the importance of careful follow-up after CAS.

## Introduction

Essential thrombocythemia (ET) is a myeloproliferative disorder presenting with sustained elevated platelets due to the chronic growth demand of megakaryocyte progenitor cells [1]. The Janus kinase 2 (*JAK2*) V617F mutation is present in 60% of ET cases, and the mutation itself may increase the risk of thrombosis [2]. However, there are few reports regarding ischemic stroke in patients with ET and the *JAK2* V617F mutation. Herein, we present the first case of late in-stent thrombosis following carotid artery stenting (CAS) in a patient with ET and *JAK2* V617F mutation presenting with symptomatic internal carotid artery (ICA) stenosis.

## Case presentation

**Figure 1:**
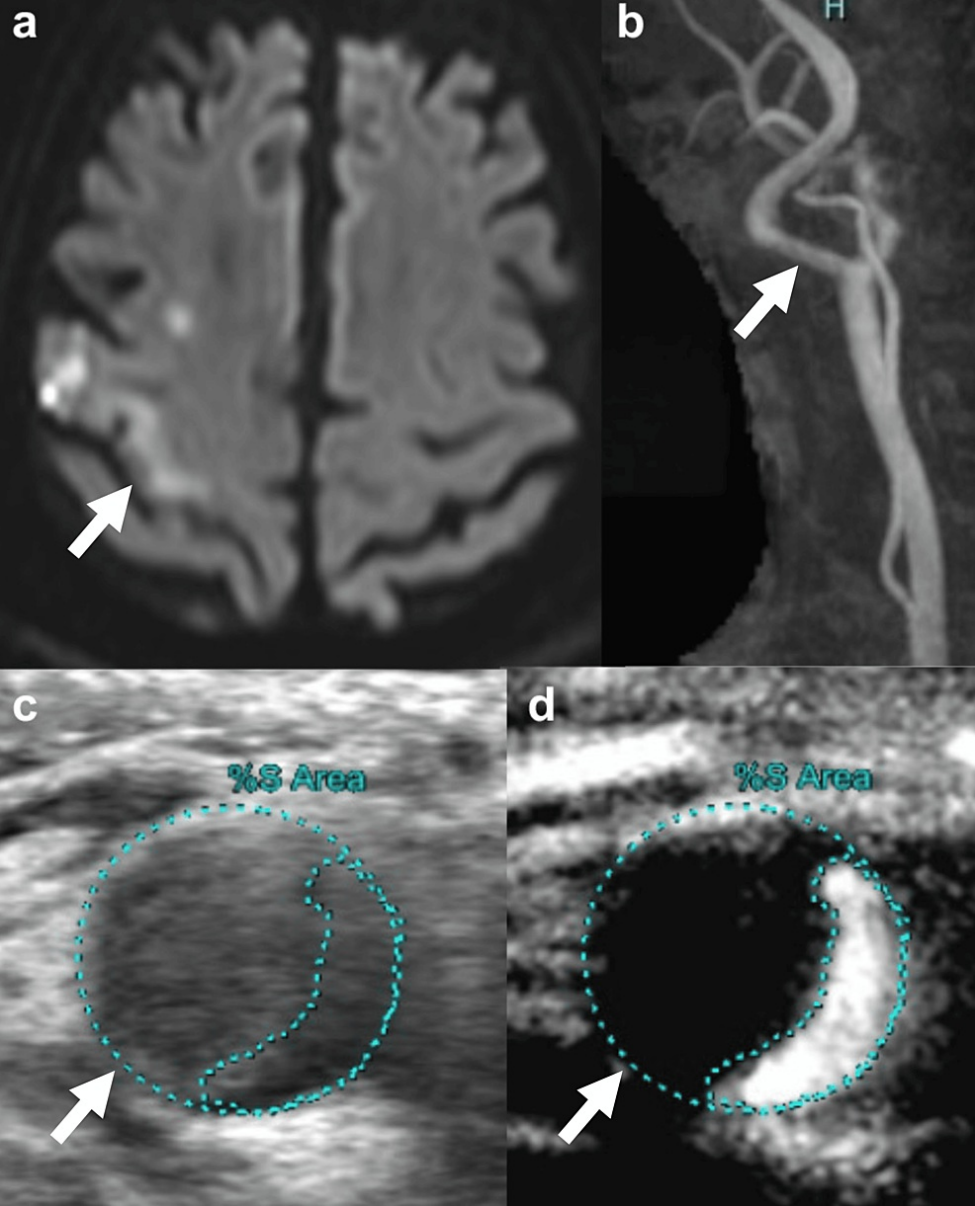
Preoperative MRI/MRA and carotid artery ultrasound (a) MRI showing hyperintensity on DWI in the right anterior central gyrus (white arrow). (b) MRA showing right ICA stenosis (white arrow). (c) Carotid artery ultrasound revealed low echoic plaque in the right ICA (white arrow). (d) Contrast-enhanced ultrasound showing a mobile plaque with stenosis (white arrow). DWI, diffusion-weighted imaging; ICA, internal carotid artery; MRA, magnetic resonance angiogram

An 80-year-old man with the *JAK2* V617F mutation was admitted to the Hematology Department due to ET which was incidentally discovered by an increase of platelets in a medical examination. He had surgery for a pituitary tumor 15 years ago but had no other medical history. Treatment was administered (hydroxyurea, 500 mg), and aspirin (ASA) was discontinued due to nasal bleeding. He presented with a three-month history of left hemiparesis and numbness. Head magnetic resonance imaging (MRI) revealed multiple acute cerebral infarctions scattered in the right frontal and parietal lobes; neck magnetic resonance angiogram (MRA) indicated right ICA stenosis (Figures 1a-1b). Following admission, he received treatment with cilostazol (CLZ; 200 mg/day), clopidogrel (CLP; 75 mg/day), and argatroban (60 mg for the first two days and 20 mg/day after the third day for five days). Carotid artery ultrasound revealed a mobile plaque in the right ICA (Figures 1c-1d). Black-blood MRI suggested an unstable plaque primarily composed of lipids due to high signal intensity in the plaque.

**Figure 2:**
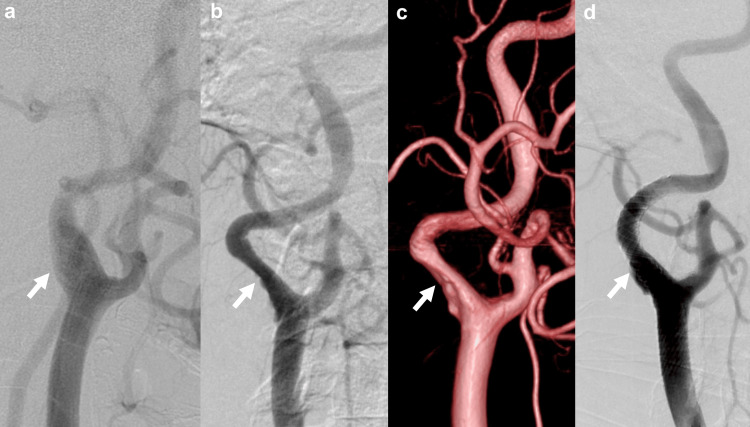
Preoperative and postoperative digital subtraction angiography (a) Angiography of the right ICA performed before surgery for a pituitary tumor 15 years ago showing no lesion (white arrow). (b) Angiography of the right ICA before CAS showing stenosis with ulcers (NASCET 41% stenosis), which was markedly progressive stenosis compared to the findings 15 years prior (white arrow). (c) 3D-DSA showing the flattening of vessels at the origin of the ICA (white arrow). (d) Angiography of the right ICA after CAS showing no residual stenosis and no thrombosis (white arrow). ICA, internal carotid artery; CAS, carotid artery stenting; NASCET, North American Symptomatic Carotid Endarterectomy Trial; DSA, digital subtraction angiography

Right ICA angiography revealed 41% stenosis (North American Symptomatic Carotid Endarterectomy Trial criteria) with an ulcer in ICA origin, which was markedly progressive stenosis compared to the findings 15 years ago (Figures 2a-2c). His platelet count was mildly elevated at 485,000/μL, and after consultation with the Hematology Department, hydroxyurea was not increased. Despite continued antiplatelet therapy, no reduction in plaque size was observed. We considered surgical treatment because of the high risk of recurrent cerebral infarction. However, carotid endarterectomy (CEA) under general anesthesia is considered high risk due to pulmonary hypertension. Therefore, the patient underwent carotid artery stenting (CAS) using a CASPER Rx 6.0 mm×30 mm stent (MicroVention, Aliso Viejo, CA, USA) and PRECISE 8.0 mm×30 mm stent (Cordis, Miami, FL, USA) with heparin infusion one month later (Figure 2d). Four months postprocedure, carotid artery ultrasound showed good stent patency without significant stenosis or thrombus, and we changed CLZ monotherapy.

**Figure 3:**
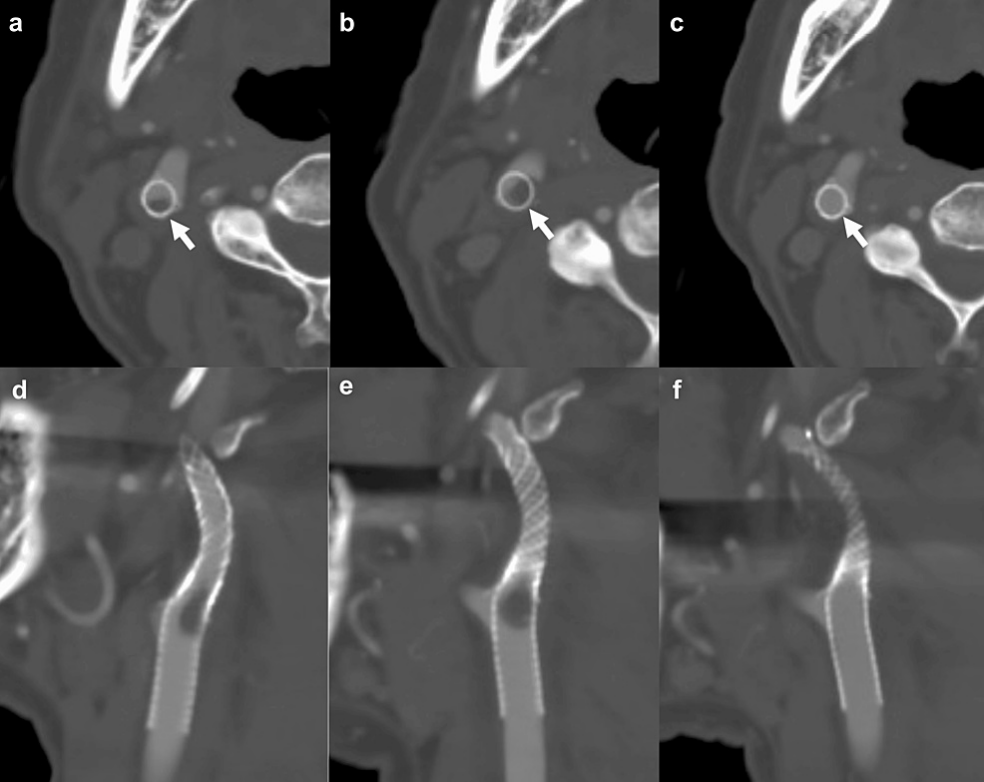
Chronological change in in-stent thrombosis in the right ICA (a), (d) CTA in the axial and sagittal plane four days after admission shows in-stent thrombosis at the origin of the right ICA (white arrow). (b), (e) CTA in the axial and sagittal 11 days after admission shows increasing in-stent thrombosis despite the addition of heparin (white arrow). (c), (f) CTA in the axial and sagittal plane 25 days after admission shows the disappearance of in-stent thrombosis due to increasing hydroxyurea dosage and the administration of aspirin (white arrow). ICA, internal carotid artery; CTA, computed tomography angiography

However, five months after the procedure, the patient suddenly experienced speech difficulties and was transported by ambulance. Head MRI did not reveal any evident new cerebral infarctions, and MRA indicated good flow in the intracranial vessels. Nevertheless, carotid artery ultrasound suggested possible thrombus formation within the stent, prompting further investigation and treatment. The Verify Now assay (Werfen, Bedford, MA, USA) indicated a P2Y12 reaction unit of 77 (baseline, 177; inhibition, 56%) with an effective antiplatelet response to CLZ. Computed tomography angiography (CTA) revealed thrombosis on the back side of the stent at the origin of the ICA (Figures 3a-3d). Then, he received treatment with CLZ (200 mg/day) and ozagrel (160 mg/day). Moreover, heparin infusion (10,000 units/day) was initiated on day 7 of hospitalization to address the possibility of secondary thrombosis. Although the activated partial thromboplastin time remained at approximately twice the reference value, the intrastent thrombus worsened on CTA (Figures 3b-3e). His platelet count increased to 567,000/μL with significant stenosis, prompting the Hematology Department to increase the hydroxyurea dosage (1000 mg/500 mg alternate-day dosing) to normalize the platelet count. The platelet count decreased to 397,000/μL, and CTA on day 25 of hospitalization indicated plaque disappearance (Figures 3c-3f).

**Figure 4:**
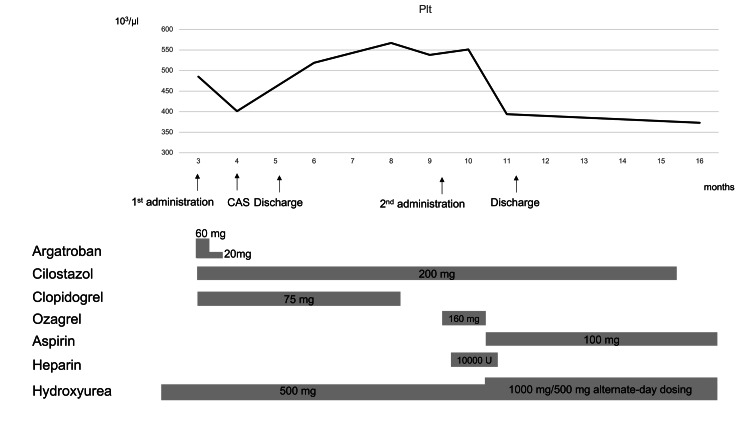
A diagrammatic representation of the treatment and platelet count in this case Plt, platelet; CAS, carotid artery stenting

There was no recurrence of cerebral infarction, and the patient was discharged on day 35 (modified Rankin scale 1). Six months after discharge, no intrastent thrombus or cerebral infarction recurrence was observed with ASA (100 mg/day) and hydroxyurea (Figure 4).

## Discussion

ET is an infrequent clonal disorder of hematopoietic stem cells. ET manifests as thromboembolism in 13% of cases, with an annual recurrence rate of 5.6% [3]. Thrombosis is the primary precipitating factor leading to mortality in individuals afflicted with ET [4]; previous investigations have indicated a predominance of cerebrovascular thrombotic events [1]. Moreover, our patient presented with the *JAK2 *V617F mutation, identified in 60% of ET cases [2]. Recently, the *JAK2 *V617F mutation itself has also been reported to increase the risk of thrombosis. There is a scarcity of literature concerning the occurrence of ischemic stroke in patients with ET and the *JAK2 *V617F mutation; thus, there is an unclear understanding of the natural progression and specific therapeutic strategies. This represents the inaugural instance of late in-stent thrombosis following CAS in a patient with ET and the *JAK2* V617F mutation, presenting with symptomatic ICA stenosis.

The presence of the *JAK2 *V617F mutant gene is an independent factor for a 2-3-fold increase in the incidence of thrombosis among patients with ET [5]. Moreover, it has been linked to both arterial and venous thrombosis, as well as an escalated susceptibility to cardiovascular and cerebrovascular ailments, encompassing the development of coronary heart disease and stroke [6]. Santilli et al. reported that the *JAK2 *V617F-positive gene mutation is expressed in both hematopoietic stem cells and vascular endothelial cells [7]; additionally, they observed that soluble thrombomodulin, soluble selectin, and tissue factor significantly increased in patients with polycythemia vera and the *JAK2* V617F mutation when compared with patients without this mutation. Additionally, there are reports that the *JAK2 *V617F mutant gene alters erythrocyte membrane components, making them more likely to adhere to vascular endothelial cells, and that increased blood cell counts affect vascular endothelial cells, causing a fibromuscular intimal proliferation in arteries without thrombus occlusion [8]. Therefore, the mutation gene itself may impair the antithrombotic activity of vascular endothelial cells and cause vascular endothelial cell damage. 

Previous reports regarding strokes due to ET with the *JAK2 *V617F mutation included cases such as floating thrombus of the ICA [9], crescendo transient ischemic attack, and symptomatic intracranial artery stenosis or occlusion, including the ICA, middle cerebral artery [10], and vertebral artery [11]. To our knowledge, five cases, including our case, have been reported regarding cervical ICA stenosis in patients with ET and the *JAK2 *V617F mutation [12,13]. The predominant disease type observed was A to A embolism, characterized by moderate to severe stenosis.

The treatment plan for people with ET is often based on the thrombotic and bleeding risk scale [2]. Our patient was regarded as having a high risk of thrombosis as he was aged >60 years and had a history of stroke and hypertension, as well as the *JAK2 *V617F mutation. Treatment for high-risk diseases is advised to include low-dose ASA and hydroxyurea (starting dose: 500 mg BID). Hydroxyurea is known to have antithrombotic effects other than thrombocytopenia, which is thought to be achieved by suppressing leukocyte function [14]. Moreover, the combination of antithrombotic therapy and hydroxyurea has been shown to result in a lower rate of recurrent thrombosis than monotherapy [3].

To mitigate the risk of thrombotic events in patients diagnosed with ET, it is generally advisable to maintain platelet counts below 400-600×10^3^/L. However, the optimal target platelet count for preventing stroke remains uncertain. Certain instances have reported ischemic stroke occurrences at platelet counts within the range of 400-600×10^3^/L [9,12]. In our case, despite platelet counts ranging from 500-600×10^3^/L, progression of ICA stenosis was observed. Notably, some reports indicate that mobile plaque regression or complete resolution, along with enhancement of blood flow, can be achieved through the normalization of blood cell counts facilitated by the administration of antitumor medications while concurrently employing antiplatelet therapy [15]. In this case, plaque regression was also obtained with a decrease in platelets. Kwon et al. demonstrated a close relationship between inflammatory conditions and the progression of atherosclerosis in myeloproliferative disorder and that reducing inflammation may help to regress plaque [16]. Tosetto et al. indicated that platelet count is the most important factor for determining how well once-daily low-dosage ASA inhibits thromboxane A2 in ET, which may also improve the efficacy of antithrombotic therapy itself [17]. Therefore, it is necessary to suppress the disease state and normalize platelet counts, especially in progressive cases, similar to our own.

ASA may also have been appropriate for our patient based on prior studies that suggested low-dose ASA for thrombosis prevention. In our case, CLZ was selected owing to the patient's aversion to aspirin-induced nasal bleeding and to reduce restenosis as previously reported in patients undergoing CAS [18]. No studies have compared the therapeutic efficacy of CLZ and ASA in patients with ET and the *JAK2* V617F mutation; however, as previous reports have documented thromboxane metabolite excretion due to platelet activation [19], the administration of aspirin, which inhibits thromboxane A2 (TXA2), is sensible. According to Tosetto et al., platelet count appears to be the most important factor for determining how well once-daily low-dosage aspirin inhibits TXA2 in ET [17].

In our case, platelet activation may have promoted interactions with endothelial cells, monocytes, and neutrophils, which were stronger with the *JAK2 *V617F mutation, further exacerbating the inflammatory response. Since TXA2 contributes to the amplification of platelet activation by almost all platelet agonists, we think that it made sense to suppress TXA2. Therefore, platelet normalization and suppression of inflammation using hydroxyurea and ASA administration are imperative in progressive instances, such as in our case.

In numerous instances, plaque regression can be attained solely through conservative therapeutic approaches. Nonetheless, there exists a record of floating thrombosis [9], warranting the contemplation of surgical interventions such as CAS and CEA in cases where plaque regression proves unattainable. While the literature on arterioplasty for ICA stenosis in patients with ET remains scarce, a documented report highlights the successful implementation of ICA stenting in such patients. Furthermore, Au et al. illustrated a triumphant instance of emergency ICA stenting, accompanied by the administration of a platelet glycoprotein IIb/IIIa antagonist, in a patient with ET [13]. In our case, although successful ICA stenting was also achieved without complications in the acute period, in-stent thrombosis occurred in the chronic period. The abnormal platelet function and inflammatory response due to *JAK2* V617F-positive ET might promote neointimal hyperplasia, and then these functions might become more active with the reduction of antiplatelet drugs, leading to plaque formation. This highlights the importance of careful follow-up, even after CAS.

## Conclusions

ET is a rare myeloproliferative disorder complicated by thrombosis, but it can be encountered in stroke treatment. The *JAK2 *V617F mutation is sometimes present in ET cases, and the mutation itself can increase the risk of thrombosis. In cases of rapid thrombosis due to the *JAK2* V617F mutation, unlike other thromboses, control of platelets and inflammation with hydroxyurea and ASA may help improve the condition. This case also highlights the importance of careful follow-up after CAS.
